# Evaluation of the Interfaces between Restorative and Regenerative Biomaterials Used in Vital Pulp Therapy

**DOI:** 10.3390/ma14175055

**Published:** 2021-09-03

**Authors:** Maria Teresa Xavier, Ana Luísa Costa, Francisco José Caramelo, Paulo Jorge Palma, João Carlos Ramos

**Affiliations:** 1 Institute of Pediatric and Preventive Dentistry, Faculty of Medicine, University of Coimbra, 3000-075 Coimbra, Portugal; aluisacosta@sapo.pt; 2 Centre for Innovation and Research in Oral Sciences (CIROS), Faculty of Medicine, University of Coimbra, 3000-075 Coimbra, Portugal; ppalma@uc.pt; 3 Laboratory of Biostatistics and Medical Informatics, Faculty of Medicine, University of Coimbra, 3000-075 Coimbra, Portugal; fcaramelo@fmed.uc.pt; 4 Institute of Endodontics, Faculty of Medicine, University of Coimbra, 3000-075 Coimbra, Portugal; 5 Institute of Operative Dentistry, Faculty of Medicine, University of Coimbra, 3000-075 Coimbra, Portugal; joao.ramos@ipmd.pt; 6 Laboratory of Biomechanical Tests, Faculty of Medicine, University of Coimbra, 3000-075 Coimbra, Portugal

**Keywords:** vital pulp treatment, calcium silicate cements, adhesive systems, adhesion, scanning electron microscopy, pulpotomy, direct pulp capping

## Abstract

Background: Calcium-silicate-based cements (CSC) have gained an increasing scientific and clinical relevance, enabling more conservative approaches, namely pulp preservation and regeneration therapies. This research aims to study the influence of four clinical variables on the interfaces between CSC and composite adhesive restoration, concerning shear bond strength (SBS) and ultra-morphological patterns. Methods: SBS tests were performed in 320 specimens divided in 16 groups (n = 20) according to: two CSC (NuSmile^®^ NeoMTA, Biodentine^TM^); two adhesive systems (Clearfil^TM^ SE Bond 2 (CSEB2), Clearfil^TM^ Universal Bond Quick (CUBQ)); optional application of an additional hydrophobic bonding layer (HBL); two restoration times (immediate, seven days). Scanning electron microscopy (SEM) was performed to conduct the ultra-morphology interface analysis in 32 deciduous molars prepared and randomly allocated into the 16 groups. Results: Globally, SBS tests showed higher bond strength of CUBQ compared to CSEB2 (*p* < 0.001), as with an additional HBL application (*p* = 0.014) and delayed restoration (*p* < 0.001). SEM showed the interpenetration between adhesive systems and CSC forming a hybrid layer, whose depth and thickness depended on the restoration time and adhesive strategy. Conclusions: The independent clinical variables adhesive system, application of an additional HBL and restoration time affected the bond performance and ultra-morphological interface between composite adhesive restoration and CSC.

## 1. Introduction

The nomenclature used to identify materials based on tri/dicalcium silicate has confused the dental community, because terms such as bioceramic, biosilicate or bioactive endodontic cements are non-specific [1]. In general, all are hydraulic dental cements, relying primarily on hydration reactions for setting, as opposed to the more usual acid–base systems used in dentistry [2,3]. They set and are stable under water [4], do not deteriorate when employed in wet environments, reach their optimal physical and mechanical characteristics and form calcium hydroxide as a by-product of the hydration reaction [5]. 

Calcium silicate-based cements (CSC) have gained an increasing clinical relevance, enabling a more conservative approach based on pulp preservation and regeneration. To overcome some of MTA’s conventional limitations, new cements have been developed, such as NuSmile^®^ NeoMTA (NuSmile Ltd. Houston, TX, USA) and Biodentine^TM^ (Septodont, Saint-Maur-des-Fosses Cedex, France), combining the biocompatibility, bioactivity and remineralization of CSC with improved physicochemical properties (high compressive strength, excellent sealing ability, ease of handling, versatility, increased density, and fast setting time) and absence of tooth discoloration. However, there are limited in vitro and in vivo studies investigating the implications of the interaction between cements and the restorative adhesive materials that are crucial for the success of the restorative treatment; the adhesive interfaces are an important clinical factor affecting the longevity and predictability of the final restoration [6,7,8]. The characteristics of the adhesive interface (its hybridization pattern, namely micromechanical and chemical interaction) depend on the technique and type of materials used.

Therefore, this research aims to study the influence of four clinical variables on the interfaces between CSC and composite adhesive restorations, concerning shear bond strength (SBS) and ultra-morphological patterns. The tested null hypotheses were: H_0_1—there is no difference between the two CSC evaluated (NuSmile^®^ NeoMTA and Biodentine^TM^); H_0_2—there is no difference between the two adhesive systems tested (Clearfil^TM^ SE Bond 2 (CSEB2) and Clearfil^TM^ Universal Bond Quick (CUBQ)); H_0_3—there is no difference between groups with or without an additional hydrophobic resin bonding layer (HBL); H_0_4—there is no difference between groups with different times for the final restoration (immediate or delayed for seven days).

## 2. Materials and Methods

The methodology included a quantitative and a qualitative analysis of the adhesive interfaces between CSC and adhesive composite restorations, comprising SBS tests and ultra-morphological analysis by scanning electron microscopy (SEM).

### 2.1. Shear Bond Strength Tests

The sample size was calculated using the software G*Power 3.1.9.2 (University of Düsseldorf) [9]. Power calculation was conducted to determine the minimal number of teeth required for the SBS test, as the principal measure. Considering an expected mean difference of 2.0 MPa and a standard deviation of 4.5 MPa [10] with a 95% power with an alpha-type error of 0.05, a total sample size of 133 samples was reached for each main group. In the present work it was decided to perform 160 samples for each main effect comparison, making a total of 320 specimens and SBS tests, divided by 16 groups (n = 20), according the 4 independent clinical variables evaluated (Table 1).

#### 2.1.1. Specimen Preparation

Metallic blocks (30 mm height × 15 mm diameter), with a central cylindrical cavity measuring 4 mm diameter and 2 mm height, with a retentive 360° groove at the bottom of the cavity, were specifically designed and fabricated for this type of research work [10]. 

Each CSC was prepared according to the manufacturer’s instructions (Table S1). The central hole was filled with the CSC, digitally compressed with a humid cotton pellet and allowed to set. Samples from the immediate restoration groups were left for 3 min of setting time for NuSmile^®^ NeoMTA and 12 min of setting time for Biodentine^TM^, prior to adhesive/restorative procedures, while samples for the delayed restoration groups were stored for 7 days for both materials before adhesive/restorative procedures (Table 1). 

The 16 experimental groups were randomly selected for specimen preparation. Each group was prepared separately and according to the type of CSC, adhesive, additional HBL application and adhesive restoration timing (Table 1).

#### 2.1.2. Restorative Procedures

##### Groups with Immediate Restoration

After the initial respective setting time for each CSC (3 and 12 min), the adhesive systems were applied over the CSC surface according to the manufacturer’s general instructions. There are currently no specific instructions from the manufacturers for the application of these adhesives on CSC.

For the CUBQ groups, the adhesive was applied with the applicator brush and left in place for 20 s. After that, the surface was dried with air until the bond did not move and was light-cured for 10 s at “High Power” mode (Bluephase^®^ Style M, Ivoclar, Vivadent, Schaan, Liechtenstein). In groups with application of an additional HBL, an extra layer of CSEB2 Bond was applied over de adhesive, dried with a mild airflow and light-cured.

After the adhesive procedures were finished and just before its final light-curing step was performed, a gelatin cylindrical capsule (Torpac^®^ Fairfield, NJ, USA) was placed over the adhesive surface and the final 10 s light curing of the adhesive was done. After that, the capsule was incrementally filled with a flowable composite resin, SDR^TM^ Bulk-fill flowable composite (Dentsply DeTrey; Konstanz, Germany), and light-cured for a total time of 60 s with the same light cure unit.

From the CSEB2 groups, the primer was applied, left in place for 20 s and dried with mild airflow. Then, the bond was applied and distributed evenly with mild airflow and left for 20 s. The following steps were carried out as described previously for CUBQ, namely with regards to photopolymerization, placement or not of additional HBL and restorative procedures. 

##### Groups with Delayed Restoration

After placement of the CSC inside the metallic blocks, the materials were covered by glass ionomer cement (GIC) (Ionostar^®^ Molar—VOCO^®^ GmbH, Cuxhaven, Germany) and stored in an incubator at 37 °C with 100% humidity for 7 days. After the storage period, the GIC was removed with black coarse aluminum oxide abrasive discs, 3M™ Sof-Lex^TM^ (3M ESPE, St. Paul, MN, USA), until a flat surface of the CSC was exposed and polished using water sandpaper. The same adhesive and restorative procedures were applied as described for the immediate groups. A single operator carried out all the adhesive and restorative procedures. During all specimen preparations, the registered room temperature was about 23 °C, with 40% humidity.

#### 2.1.3. Shear Bond Strength Tests

**Figure 1:**
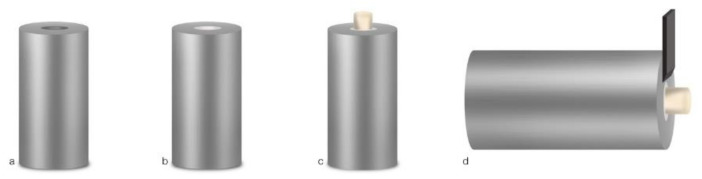
Schematic diagram of the experiment set-up showing how the samples were prepared for SBS strength testing: (**a**) cylindrical metallic blocks; (**b**) the hole in the middle was filled with the CSC; (**c**) after adhesive procedures, a sectioned gelatin capsule was applied on the surface of the CSC and filled with the flowable composite resin; (**d**) a chisel-edge plunger was mounted into the testing machine and positioned, so that the leading edge was aimed at the CSC/adhesive interface. Adapted from Altunsoy, Tanriver, et al. and from Palma et al. [10,11].

Before proceeding with the SBS tests, all samples were stored in the same incubator and conditions for 48 h. Each block was fixed in a universal testing machine (Model AG-I, Shimadzu Corporation, Kyoto, Japan), in a shear mode at a cross-head speed of 0.5 mm/min and 250 N, with a chisel-shaped rod, until failure occurred. The force registered, measured in N, was divided by the cross-sectional area of the adhesive interface and expressed in MPa. To avoid bias, a single and blinded operator carried out the SBS tests (Figure 1).

#### 2.1.4. Fracture Pattern Analysis

The fractured surfaces of each sample were examined under a stereomicroscope (Opmi Pico, Carl Zeiss Surgical, Oberkochen, Germany) equipped with a halogen light source and a global magnification of 21.3×. The specimens were classified according to the failure modes [12,13,14]: adhesive fracture, cohesive fracture exclusively in the CSC, cohesive fracture exclusively within the restorative material, mixed fracture (comprises both adhesive and cohesive fracture). 

#### 2.1.5. Statistical Analysis

The SBS test results were described using mean, standard deviation and minimum and maximum values. The normality of data distribution testing was carried out using the Shapiro–Wilk test. A four-way ANOVA was conducted to compare the main effects (type of CSC, adhesive system, presence or absence of additional HBL and timing of restoration). The interaction between different combinations of effects was evaluated with a descriptive table for each group generated by the conditions analyzed. The association between the fracture type and the CSC, adhesive system, presence of HBL and restoration time was assessed using Fisher’s exact test. The Bonferroni correction was used to adjust for multiple comparisons. Two-tailed *p* values were calculated with a significance level set at α = 0.05. Statistical analysis was performed using IBM SPSS^®^ version 26 software (Chicago, IL, USA). The significance level was set at α = 0.05. 

### 2.2. Bond Interface Evaluation by Scanning Electron Microscopy

#### 2.2.1. Specimen Preparation

From a pooled biobank of extracted teeth, 32 deciduous molars with at least one third of the root and without furcation involvement were randomly selected. Before the extraction, the patients and their parents were informed about the use of the teeth for research or educational purposes and their informed consent was obtained. Because the samples used were collected from a pooled biobank, they are categorized as “irreversibly anonymized” (approval was obtained from Ethics Committee of Faculty of Medicine, of the University of Coimbra, Ref 002-CE-2020-020). Extracted teeth were stored in an aqueous chloramine solution 0.5%, at 4 °C for up to 6 months, following the norm ISO/TS 11405:2015, which was renewed every month.

Occlusal cavities exposing the pulp chamber were made in each tooth and the remaining pulp tissue was removed with a spoon excavator, rinsed with sterile saline solution and air-dried. These teeth were mounted in self-cure acrylic resin blocks that the cement-enamel junction was flush with the resin surface. The teeth were randomly allocated into 16 groups (n = 2), following the same variables described for SBS tests.

#### 2.2.2. CSC Placement and Adhesive/Restorative Procedures

The CSC were placed into the pulp chamber cavity, allowed to set, adhesively treated, restored and stored as described previously for the same 16 groups evaluated by SBS tests. After storage for 1 week, the teeth were multi-sectioned in a buccolingual direction along their longitudinal axis using a high-precision diamond cut-off wheel from a high-precision machine (Accutrom 50 machine, Struers, Denmark) with approximately 1000 μm thickness.

The specimens were polished and treated by 35% phosphoric acid gel for 15 s, followed by washing and drying. They were sequentially dehydrated in increasing concentration of ethanol (50%, 75%, 95%, 100%) and were mounted on aluminum stubs, sputter-coated with gold–palladium and observed by field-emission SEM (Hitachi S-4100, Tokyo, Japan) at various magnifications.

## 3. Results

### 3.1. Shear Bond Strength Tests

#### 3.1.1. Main Effects of Independent Variables

Concerning the main effects of independent variables on shear bond strength, the normality of data distribution testing was carried out using the Shapiro–Wilk test and the normality assumption was violated. Since the number of samples between groups for main effect analysis was similar (n = 160) and ANOVA is considered a robust test against normality violation, a four-way ANOVA analysis was conducted to establish statistically significant differences between the main effects (CSC, adhesive system, additional HBL and restoration time) as well as their interaction effects on the SBS test results. 

Overall, a statistical significant difference was found in the ANOVA test for the mean SBS among the tested groups for the main effects of four independent factors: F(4. 315) = 13.112, *p* < 0.001.

- Main effect “type of CSC”

No statistically significant difference was found in the mean SBS values between Biodentine^TM^ and NuSmile^®^ NeoMTA (*p* = 0.897) (Table 2). To test the interaction effects of the other independent variables, a comparison between all the groups with Biodentine^TM^ and NuSmile^®^ NeoMTA was done keeping the same variable combination (restoration timing, adhesive system and number of HBL). Only the combination of CSEB2 with an extra HBL and immediate restoration was statistically different between the two CSC materials, with higher bond strength for Biodentine^TM^ (Table 3 and Table 4).

- Main effect “type of adhesive system”

CUBQ showed statistically higher SBS values than CSEB2 (*p* < 0.001) (Table 2). The interaction effect of the other independent variables (CSC type, presence of additional HBL and restoration time) revealed that only the combination of NuSmile^®^ NeoMTA with no extra HBL and delayed restoration was statistically different between the two adhesive systems with higher SBS for CUBQ (Table 3 and Table 4).

- Main effect “additional hydrophobic resin layer”

The application of an additional HBL resulted in a significant higher mean SBS value compared to no additional HBL application (*p* = 0.014) (Table 2). Results between the groups with or without an additional HBL concerning the other remaining independent variables (CSC, adhesive systems and restoration timing) revealed that although 5 of the 8 comparison values were higher with the presence of an HBL, only the combination BiodentineSE0I was statistically different from the combination BiodentineSE1I (Table 3 and Table 4).

- Main effect “timing of the definitive restoration”

Concerning the different restoration times, the delayed group showed statistically higher mean SBS values than the immediate one (*p* < 0.001) (Table 2). The interaction effect of the other independent variables revealed that the restoration timing was only significant for the combination NuSmile^®^ NeoMTA, CUBQ without an additional HBL, showing better results for delayed restoration (Table 3 and Table 4). 

#### 3.1.2. General Distribution of Shear Bond Strength Results between All Groups 

A descriptive analysis and dispersion graph were done to overview all the groups. Table 2 shows the mean, standard deviation, median, interquartile range, minimum and maximum value and Table 3 the *p*-values from direct comparison between groups. 

From all the tested groups, the NeoMTAU07 showed the highest mean SBS value (11.36 ± 5.72), followed by the NeoMTAU17 (10.44 ± 4.65), with no statistically significant difference between them (*p* > 0.05). The highest mean SBS value in the Biodentine^TM^ group was BiodentineU07 (9.44 ± 4.58), with no statistically significant difference between this group and NeoMTAU07. 

The CUBQ revealed better bond performance in the NuSmile^®^ NeoMTA group (*p* < 0.05), compared to CSEB2. 

No application of an extra HBL, independently of the timing of the restoration (immediate or after seven days), resulted in a weaker bond for Biodentine^TM^ and NuSmile^®^ NeoMTA combined with the CSEB2, with statistically significant differences between NuSmile^®^ NeoMTA, CSEB2, delayed restoration with and without HBL.

The group BiodentineU07 (9.44 ± 4.58) revealed the best performance within the Biodentine^TM^ group. The BiodentineSE0I revealed the weakest performance.

#### 3.1.3. Fracture Pattern Analysis

The same examiner repeated the evaluation of fracture pattern one month after the initial evaluation, re-scoring 32 specimens. A Kappa coefficient of 0.808 (*p* < 0.001) was found representing a strong agreement between the two analyses. The fracture pattern was compared between the groups regarding the four main effects (Table 2). According to Fisher’s exact test there was no statistically significant association between the fracture type and the CSC used (*p* = 0.127), with more cohesive fractures in both groups. There was a statistically significant difference between the adhesive systems (*p* < 0.001). Although both adhesive systems presented more cohesive fractures, CSEB2 showed more adhesive fractures than CUBQ. Conversely, CUBQ showed more mixed failures than CSEB2. We found a statistically significant association between groups with and without an application of an extra HBL (*p* = 0.011). In the group with an additional HBL, the more prevalent fracture was cohesive in the CSC, followed by adhesive fracture. In the group without an additional HBL, the more prevalent fracture was also cohesive but followed by mixed pattern. A statistically significant association was verified between the fracture pattern and the timing of restoration (*p* < 0.001). The delayed restoration group had more adhesive failures compared with the immediate group. Conversely, the immediate restoration had more cohesive failures in the CSC.

**Figure 2:**
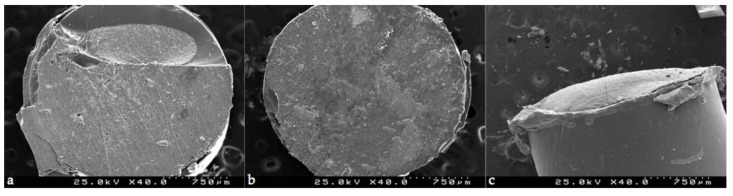
Scanning electron microscope images of mixed fracture (**a**), adhesive fracture (**b**) and cohesive fracture in CSC (**c**) (original magnification 40×).

Representative SEM images of specimens with three different patterns of failure between resin composite and CSC are presented (Figure 2).

A total of 32 specimens (10% of the sample) were randomly selected and reanalyzed to determine the intra-examiner reproducibility. 

### 3.2. Bond Interface Evaluation by SEM

**Figure 3:**
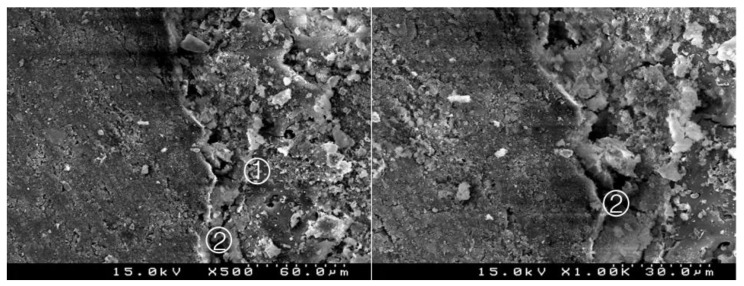
Group 1 (BiodentineSE0I): straight interdiffusion of the adhesive material protruding into the CSC. Cement particles are involved by the adhesive; a CSC/adhesive hybrid layer is observed ① with some empty spaces on the top of the hybrid layer ② (original 500× and 1000×).

**Figure 4:**
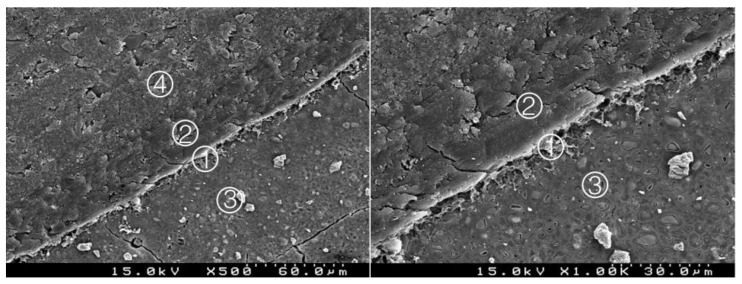
Group 2 (BiodentineSE07): hybrid layer with some empty spaces corresponding to the removed inorganic superficial content of the CSC and some deeper content of the adhesive. A remaining organic mesh of the adhesive in the hybrid layer is shown ①. Adhesive ②, Biodentine^TM^ ③, composite resin ④ (original magnification 500× and 1000×).

**Figure 5:**
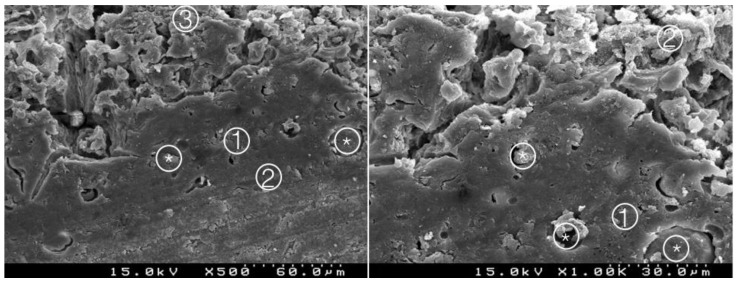
Group 3 (BiodentineSE1I): considerable interdigitation between the adhesive system and CSC. A thick hybrid layer is presented ①. Particles of cement involved by the adhesive (*). Adhesive ② and Biodentine^TM^ ③ (original magnification 500× and 1000×).

**Figure 6:**
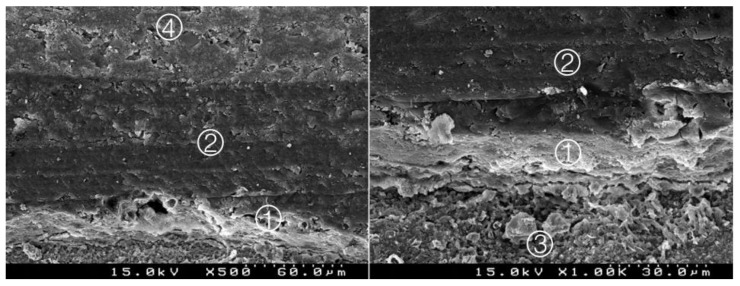
Group 4 (BiodentineSE17): some interpenetration between the adhesive system and CSC. A less deep hybrid layer is observed ① between the adhesive with a thick layer ② and the Biodentine^TM^ ③. Composite resin ④ (original magnification 500× and 1000×).

**Figure 7:**
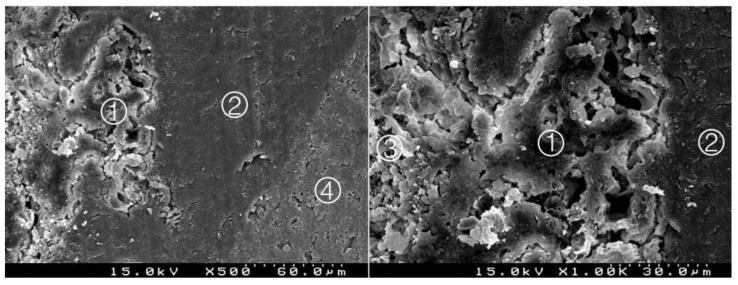
Group 5 (BiodentineU0I): deep interpenetration between the adhesive and the cement, with particles of cement involved by the adhesive. A thick hybrid layer is presented ① between the adhesive ② and the Biodentine^TM^ ③. Composite resin ④ (original magnification 500× and 1000×).

**Figure 8:**
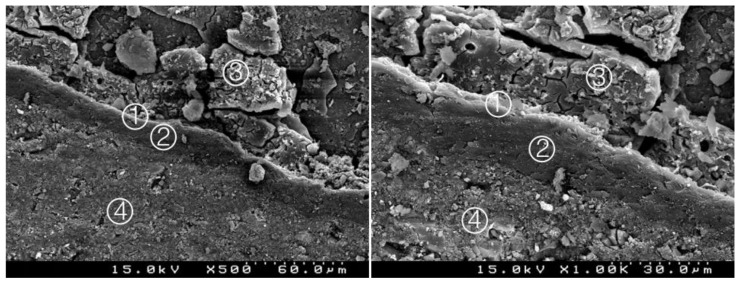
Group 6 (BiodentineU07): some interdigitation between the adhesive and cement. A less deep hybrid layer is observed ① between the adhesive ② and the Biodentine^TM^ ③. Composite resin ④ (original magnification 500× and 1000×).

**Figure 9:**
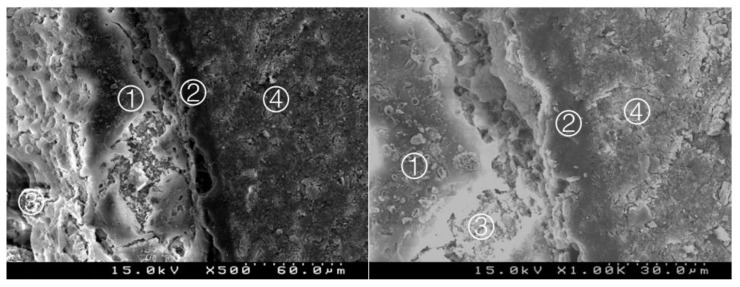
Group 7 (BiodentineU1I): deep interdigitation between the adhesive and the cement, with particles of cement involved by the adhesive. A thick hybrid layer is presented ① between the adhesive ② and the Biodentine^TM^ ③. Composite resin ④ (original magnification 500× and 1000×).

**Figure 10:**
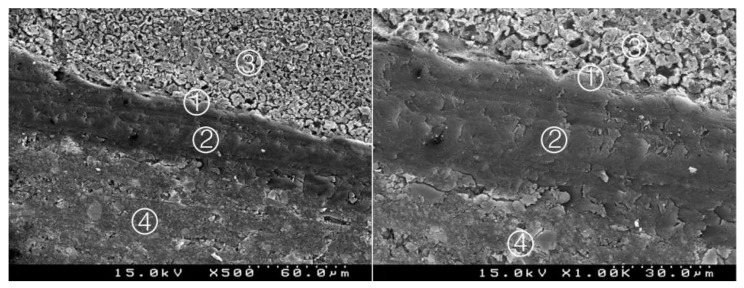
Group 8 (BiodentineU17): less interpenetration between the adhesive and the cement. A less deep interdiffusion layer is presented ① between the adhesive in a thick layer ② and the Biodentine^TM^ ③. Composite resin ④ (original magnification 500× and 1000×).

**Figure 11:**
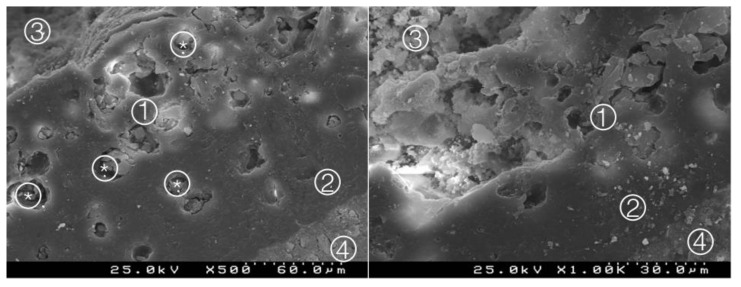
Group 9 (NeoMTASE0I): deep interdiffusion between the adhesive and the cement, with particles of cement involved by the adhesive (*). A thick hybrid layer is observed ① between the adhesive ② and the NuSmile^®^ NeoMTA ③. Composite resin ④ (original magnification 500× and 1000×).

**Figure 12:**
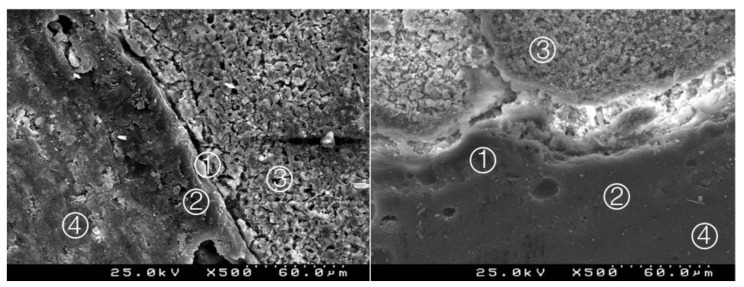
Group 10 (NeoMTASE07): less deep interdigitation between the adhesive and the cement. A thin interdiffusion layer is observed ① between the adhesive ② and the NuSmile^®^ NeoMTA ③. Composite resin ④ (original magnification 500× and 1000×).

**Figure 13:**
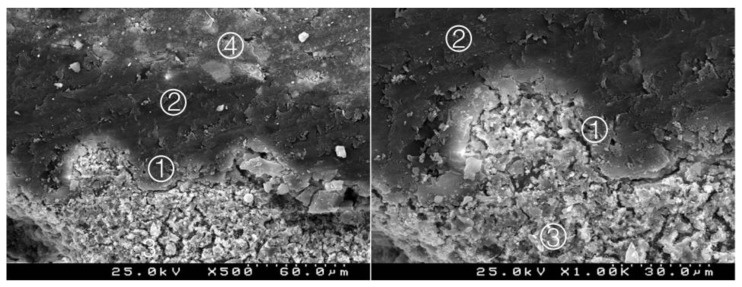
Group 11 (NeoMTASE1I): hybrid layer ① with evident interpenetration between the adhesive ② and the NuSmile^®^ NeoMTA ③. Composite resin ④ (original magnification 500× and 1000×).

**Figure 14:**
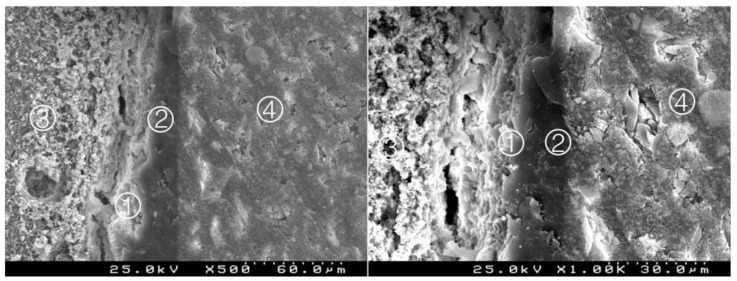
Group 12 (NeoMTASE17): some interdigitation between the adhesive system and CSC. A less deep or thinner interdiffusion layer is observed ① between the adhesive ② and the NuSmile^®^ NeoMTA ③. Composite resin ④ (original magnification 500× and 1000×).

**Figure 15:**
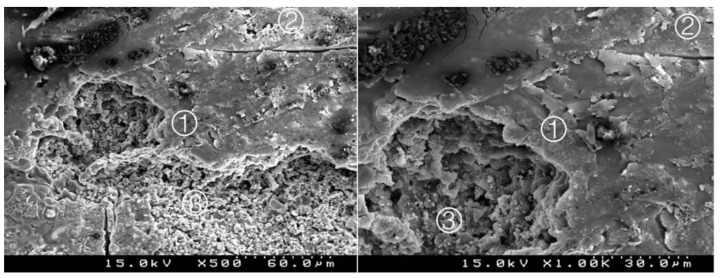
Group 13 (NeoMTAU0I): interpenetration between the adhesive and the cement, with particles of cement involved by the adhesive. A thick interdiffusion layer is presented ① between the adhesive ② and the NuSmile^®^ NeoMTA ③ (original magnification 500× and 1000×).

**Figure 16:**
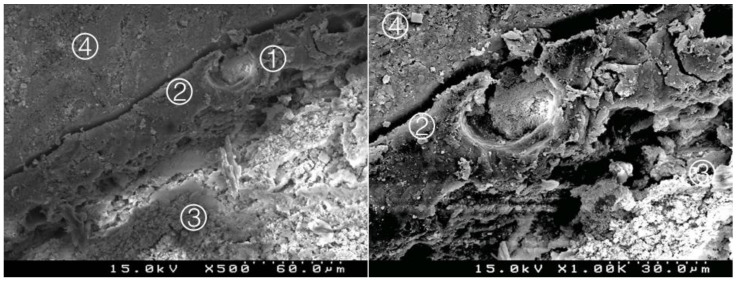
Group 14 (NeoMTAU07): interdigitation between the adhesive and cement. An interfacial gap and a less deep interdiffusion layer are observed ① between the adhesive ② and the NuSmile^®^ NeoMTA ③. Composite resin ④ (original magnification 500× and 1000×).

**Figure 17:**
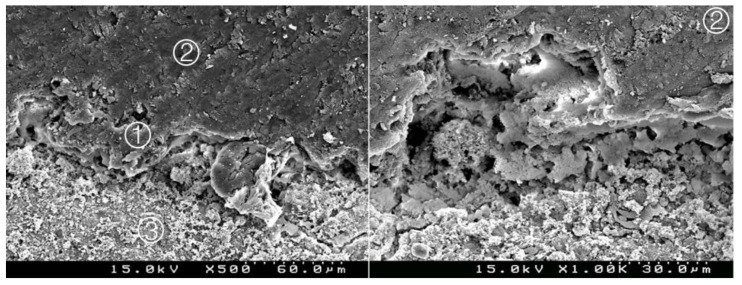
Group 15 (NeoMTAU1I): deep interdigitation between the adhesive and the cement. A thick hybrid layer is presented ① between the adhesive ② and the NuSmile^®^ NeoMTA ③ (original magnification 500× and 1000×).

**Figure 18:**
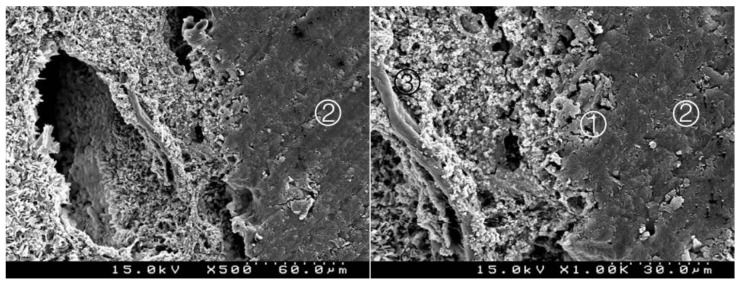
Group 16 (NeoMTAU17): less interdigitation between the adhesive and the cement and an interfacial gap on the hybrid layer. A less deep interdiffusion layer is presented ① between the adhesive layer ② and the NuSmile^®^ NeoMTA ③ (original magnification 500× and 1000×).

Generally, in all specimens interpenetration between the CSC and the adhesive systems was present, forming a hybrid layer or interdiffusion zone between adhesive and CSC. Its thickness and depth vary according to the timing of restoration and adhesive procedure. In the delayed restoration groups, this interpenetration was less deep than in the immediate groups. The pattern of the morphological interaction varies depending on the adhesive procedure and the time of restoration. In the CSEB2 and in immediate groups the superficial “dissolution” of the CSC and incorporation of particles into the adhesive layer was generally greater, as well as the adhesive filling of spaces between the inorganic content of the CSC. The thickness of the adhesive layer varies according to the adhesive procedure; it was thicker in groups with an additional HBL. Some cracks and interfacial gaps observed are related to artifacts due to technical preparation for SEM observation, primarily in the cutting and dehydration process (Figure 3, Figure 4, Figure 5, Figure 6, Figure 7, Figure 8, Figure 9, Figure 10, Figure 11, Figure 12, Figure 13, Figure 14, Figure 15, Figure 16, Figure 17 and Figure 18). 

## 4. Discussion

SBS is a common in vitro method to analyze the performance of an adhesive system to restorative material. It includes quantitative analysis to predict the load capacity and longevity of the bonding and qualitative screening tests, and is also used to study bonding interfaces and bonding failures. Academically, the SBS is defined as the interfacial adhesion between the substrate and the bonded material, intermediated by an adhesive layer [15]. 

The shear and microtensile are the most currently used, particularly the microtensile [16]. Because CSC are brittle, in thin cross sections it must be used in bulk to avoid damage. The shear tests allow simpler specimen preparation with a reduced risk of sample damage [7]. This test was chosen for the present study and the methodology followed previous research [8,10,17].

In the published literature, the diameter and depth of the central cavity of the metallic mold differs between 3–5 mm and 1.5–2 mm, respectively [10,13,18,19,20]. Considering the studies abovementioned, in particular Palma et al. [20], which related the high frequency of cohesive fracture patterns within CSC with the adhesive area, it was decided to use central holes of 4 mm/2 mm, with a 360° deep groove, allowing better retention of the CSC.

The use of gelatin capsules may have contributed for the zero premature failures because it eased composite insertion and capsule removal, after storage in 100% humidity, not causing pressure or stress in the sample adhesive interface. Regarding the composite block dimension, many studies deployed similar sizes, such as Carretero et al. [21]; it had a diameter of 2.26 mm and a length of 3 mm. 

The sixth and seventh generation adhesives are useful in pediatric dentistry, particularly with behavior management, by reducing procedure time, simplifying multi-step etch-and-rinse procedures and minimizing technical sensitivity [22]. A new multimode generation has changed the traditional adhesive protocol, using either an etch-and-rinse or self-etch systems and with an immediate clinical performance equivalent with that of gold-standard etch-and-rinse and self-etch reference adhesive systems, Optibond^TM^ FL (Kerr, Orange, CA, USA) and CSEB2, respectively. Recently, universal adhesives with a ‘quick and flexible bonding’ concept were introduced, claiming that the waiting time to guarantee its interaction with dentin, the solvent evaporation, is no longer needed. CUBQ is a ‘no-wait’ universal adhesive, although the manufacturer’s instructions were not followed since the long-term clinical performance still needs to be proven [22,23]. 

New CSC formulations vary from traditional MTA, including finer particle sizes, which increase the surface area for faster hydration, shortening the setting time and improving handling characteristics [24]. NeoMTA**^®^** dry powder has a more regular structure, with smaller (10 μm or less) spherical particles [25,26]. Biodentine^TM^ has particles between 1–10 μm [27]. This affects the adhesion of cements to dentin by enhancing its interpenetration with it. Furthermore, it is believed that during setting the small particles lead to a decrease in the material’s porosity and an increase in its compressive strength [28]. NeoMTA Plus**^®^** and Biodentine^TM^ have similar particle size and are smaller compared to conventional MTA, which may be the reason for the first null hypothesis was not rejected.

Isolated SBS values cannot be used to draw absolute conclusions or be compared with other data; only relative study outcomes are a valid basis for further interpretation of the results [29]. Furthermore, it is difficult to compare results obtained in other studies due to the variation of a several relevant parameters (restorative materials, adhesive systems and technical application, waiting and restoration time) [15] and in the experimental methods (speed of load and magnitude of maximum load when measuring SBS) [30]. The results from the limited data concerning adhesion of restorative materials to Biodentine^TM^ reported that the methacrylate-based composites could achieve optimal SBS values (17.7 ± 6.2 MPa) [18] and with different adhesive systems varied between 15–19 MPa [13].

On the other hand, SBS of X-tra base (Voco GmbH, Cuxhaven, Germany) or Vertise^TM^ Flow (Kerr, Orange, CA, USA) was 1.69 and 1.2 MPa, respectively; Biodentine^TM^ overlaid by the composite resin (Filtek^TM^ Z-350 XT, 3M ESPE, St. Paul, MN, USA) with universal adhesive (Single Bond Universal^TM^, 3M ESPE, St. Paul, MN, USA) was 5.67 ± 6.2 MPa and was lower than previous studies and the present study. SBS of SDR^TM^ Bulk fill flowable composite with CSEB2 Bond to Biodentine^TM^ at two different times were 5.49 ± 4.28 and 6.98 ± 4.51 MPa, respectively [10,11,31]. Regarding the NeoMTA, to date, we are not aware of information about these tests that allows any type of comparison. 

Regarding adhesion to CSC, it is still unknown whether a chemical bond exists on the interface [7]. Since there is no resin structure in CSC, such as NuSmile^®^ NeoMTA or Biodentine^TM^, it might be speculated that the bond is micromechanical and results from the interdiffusion and interlocking between these materials [32]. Hydrophilic characteristics of monomers in adhesive systems can facilitate interdiffusion, but secondly it can have a negative impact attending its excessive diffusion in depth and compromising the correct polymerization of the adhesive and cement setting reaction. On the other hand, the acidity of the adhesive or phosphoric acid may be buffered by the alkalinity of the calcium silicate cement [15]. 

Some authors have revealed that the adhesive systems in either SE or TE modes had no statistically significant influence on the bond strength to the composite [7,33]. Other authors have concluded that sufficient bonding performance may be obtained without an acid etching, simplifying the adhesive step (universal adhesive applied on Biodentine^TM^ showed similar bond values in self-etch and etch-and-rinse modes) [33]. 

Additionally, a two-step self-etch adhesive system (CSEB2) exhibited higher shear bond strength than one-step (Clearfil^TM^ S3 Bond) [13], which is in agreement with previous studies [34]. There is controversy concerning the efficacy of self-etch systems applied over CSC; some investigations show they provide dentin bond strength comparable with etch-and-rinse systems [35,36], whereas others observed significantly lower bond strengths [37,38]. 

The universal adhesive (Single Bond Universal^TM^, 3M ESPE, St. Paul, MN, USA) used as a self-etch showed a SBS mean of 5.66 MPa [31]. This’s in contrast with other study that used Scotchbond Universal^®^ (3M ESPE, St. Paul, MN, USA) and obtained 13.65 MPa. Both have respected the Biodentine^TM^ setting time, so these may be due to different adhesive composition or operator/technique variable [21]. 

The present protocol design considered new adhesive strategies rather the bonding agents per se. The mean SBS varied between 3.62–11.36 MPa. Concerning the main effect “adhesive system”, the use of two-step self-etch adhesive CSEB2 resulted in a weaker bond, compared with CUBQ. The combination BiodentineSE0I and NeoMTASE0I presented the lowest bond strength in the Biodentine^TM^ and NuSmile^®^ NeoMTA groups; this may be due to precocious application of the primer over CSC. Although CUBQ had significantly higher values of bond strength than CSEB2, both contain similar functional monomers. The major difference is the primer application and the thickness of the adhesive layer: the CSEB2 was 40 μm [39] and the CUBQ was 5–10 μm [23]. This characteristic does not adversely influence the bond strength, but it may cause imperfect restorations in some clinical situations [33]. 

The functional monomers are important to improve adhesive clinical performance by increasing the bond strength with teeth. The HEMA is hydrophilic and is similarly present in both adhesives. It forms a polymeric network able to stabilize the outer surface of the cement after photopolymerization and absorbs moisture to aid hydration to the CSC setting reaction [40]. However, the hydrophilic nature of many simplified adhesives is one of the most documented factors responsible for the hybrid layer degradation [29,41,42]. HEMA has a relatively high allergic potential, lower polymerization efficiency, high water uptake and reduced nanolayering by the 10-MDP. New adhesives have been marketed with lower HEMA content or even without it [22,43,44]. Its content in CUBQ is 2.5–10%, compared to CSEB2, which is 10–30% [23,45]. As a result of this reduction, water sorption is reduced and polymerization conversion improved, which seems to be reflected in the absence of bond degradation upon 6 months aging when applied in both etch-and-rinse and self-etch modes [22]. Nevertheless, the water content of the cements themselves can remain a problem for the polymerization and stability of the adhesives. 

The organic solvents that act as carriers of the monomers into the collagen fibers network in dentin and as diluents to lower the resin viscosity can also enhance the infiltration of resins into the microporosities and spaces [46]. Another potential reason explaining the superior performance of CUBQ is the better wettability of ethanol and water presented in its composition, in contrast to CSEB2, which contains only water as a solvent [47]. 

The degradation potential of resin–dentin interfaces present in the simplified one-step self-etch adhesives results from the water osmosis, from the environment, interferes with the cross-linked polymers formation and consequently a porous hybrid layer is produced because of the elution on unreacted monomers [46,48]. To bypass this drawback an additional HBL is applied over the polymerized adhesive [49,50]. Previous reports have described its improved performance and degradation prevention of the resin–dentin bonds as a result of the increasing thickness and uniformity of the adhesive layer, as well as to reduce the fluid flow across the adhesive interface [51,52]. However, this method has not been tested with self-etch adhesive systems applied over CSC in order to evaluate the bond strength and interface structure between them. In the present study and concerning the main effect “application of an additional HBL”, the overall analyses showed that this procedure significantly increases the SBS values. 

For both CSC, the setting time is shorter than for MTA and bonding the final restoration directly after mixing the calcium silicate cement is worthwhile, as this is easier and less time consuming. However, the quality and durability of the adhesive bond between CSC and the filling material is clinically important for the longevity and predictability of the final restoration [7]. Therefore, a higher level of CSC setting is necessary before the restoration is done, since the durability of bonding may be affected by the state of the calcium silicate cement (set or unset) and the curing shrinkage of the composite may stress the unset calcium silicate cement [15]. 

In this study, restorations done after seven days exhibited better bond performance than restorations done immediately. In agreement with this, Kaup et al. reported a significant increase in the shear bond values of Biodentine^TM^ to permanent dentin between 2 days and 1 week storage times compared to that of MTA [53]. 

Concerning Biodentine^TM^ failure pattern analysis, a cohesive pattern within CSC might reflect its low cohesive resistance compared to high bond strength [20]. The literature is scarce and without consensus [21]; some studies described more cohesive fractures [13,20,31]; Tulumbaci et al. [54] found mostly adhesive failures and Altunsoy et al. [11] did not have adhesion failures. In accordance with the literature, both CSC presented similar rate of cohesive failures, but with no statistically significant association between the fracture type and the CSC used. 

To achieve a successful restorative treatment with two materials with different characteristics there should be an appropriate bond on the interface to guarantee the long-term success [55]. In a simplistic analysis, the bond is considered acceptable when cohesive fracture happens rather than adhesive [56]. However, regarding the interfacial adhesion, if the adhesive procedures significantly interfere with the cohesive properties of the substrates, the assumption of satisfactory results based on cohesive fracture patterns is not applied. 

Failure mode analysis showed a greater number of samples exhibiting more cohesive fractures in CSC in both adhesive materials, followed by adhesive fractures in CSEB2 and mixed fractures in CUBQ, with a statistically significant difference between the two adhesives. Regarding the application of an extra HBL, there was a statistically significant association between the fracture pattern and the application of an extra HBL. Furthermore, a statistically significant association was verified between the fracture pattern and timing restoration. 

Similar to our results, Palma et al. [20] found that the cohesive pattern was mostly present in the immediate group, whereas the adhesive failure had a higher rate in the delayed group. Çolak et al. [19] also described the cohesive pattern as the most prevalent, after the samples were stored in distilled water for a period of 24 h. This is in contrast with 70% of mixed fractures after 12 min [15] and may be due to fact that the specimens were stored for 28 days after the restorative procedure to guarantee a complete setting of the CSC before SBS testing. 

Altunsoy et al. [11] applied the composite resin after 72 h over the Biodentine^TM^ and did not find any adhesive fractures, but instead found cohesive or mixed. After 24 h, Deepa et al. [31] found 60% cohesive and 40% adhesive fractures, like Tulumbaci et al. [54] had mainly adhesive fractures. 

Unlike most studies that performed SBS tests immediately after the restoration, in this research and to avoid premature cohesive fractures within the incompletely set CSC, the tests were performed 48 h after. However, the cohesive pattern was the most prevalent in the immediate group. 

In the SEM from all the specimens, the interpenetrations between the CSC and the adhesive systems were presented, forming a hybrid layer or interdiffusion zone. Its thickness and depth vary in accordance with the timing of restoration and adhesive procedure. The thickness was higher in groups with an additional layer of hydrophobic resin; in the delayed groups this interpenetration was more regular and less deep than the immediate groups; we hypothesized this may be due to the presence of water from the unset CSC and hydrophilic nature of the adhesive systems.

The pattern of morphological interaction of the adhesive with the CSC was also affected by these two variables. In the CSEB2 and in the groups with immediate restoration, the superficial “dissolution” of the CSC and incorporation of particles into the adhesive layer was commonly more evident, as well as the adhesive filling of spaces between the inorganic content of the CSC. Some of these spaces were probably observed in SEM to be empty due to a possible wash-out effect of the adhesive, and even CSC particles during the preparation of the cuts for observation. 

Overall, within the limitations of an in vitro study, these findings permit us to predict the regenerative and restorative performance of CSC and the adhesive systems and emphasize the importance of an adequate choice of materials and techniques in order to optimize the clinical procedures. In addition, they highlighted new problems and issues, with potential clinical implications, which can and should be evaluated by new and different studies.

One of the limitations of the present study is the fact that the restorations aging process was not performed. Furthermore, the chemical composition of the hybrid layer was not performed to confirm the interpenetration on the interface of the CSC and restorative adhesive composite. Therefore, scanning electronic microscopy and/or energy dispersive X-ray analysis should be considered in future studies.

The laboratory studies permit us to predict the restorative performance of CSC and the adhesive systems. The influence of different variables suggests the necessity for additional research under thoroughly controlled experimental conditions. In this study, the total setting time of CSC was not considered; future studies should observe the influence of setting on the adhesion to the restorative material by evaluating the effect of allowing more time between the application of Biodentine^TM^ and NuSmile^®^ NeoMTA and the definitive adhesive restoration.

Further studies should also include CSC with different thickness, reproducing the different types of VPT, from the thin layers used in small direct pulp capping, to 2–3 mm applied in the pulpotomy. Furthermore, since this is a radiopaque material, it would be interesting to evaluate the light penetration from the UV into cement and how deeply is the adhesive polymerized. Complementarily to the knowledge from the underlying mechanisms of the adhesion to CSC resulting from microscopy imaging, the molecular interactions at deeper layers should also be assessed, in order to understand how the interlocking relation and the deeper penetration of the adhesive monomers into CSC may interfere with the biological properties of these materials, namely their biocompatibility and dentinogenic effect.

By carrying out in vivo studies, all possible micromechanical properties of CSC and adhesive systems may be investigated, accounting for their interaction and host conditions, particularly in the interfaces with dentin and pulp tissues. Thereby, it would be possible to disclose which therapeutic strategy is truly reliable for the restoration of VPT.

Later, clinical trials remain the ultimate way to collect scientific evidence on the clinical efficacy of these regenerative and restorative treatments.

## 5. Conclusions

Concerning the objectives initially defined we can conclude that, globally and with the exception of the calcium-silicate-based cement type, the remaining three clinical variables studied (type of adhesive, effect of placing an additional hydrophobic resin layer and time taken to perform the definitive restoration) can significantly affect the shear bond strength to calcium-silicate-based cements, in particular:

- The shear bond strength to Biodentine^TM^ and NuSmile^®^ NeoMTA was similar.
- Clearfil^TM^ Universal Bond Quick provided higher shear bond strength when compared to Clearfil^TM^ SE Bond 2.
- The application of an additional hydrophobic resin layer over the adhesive improved the shear bond strength of composite adhesive restoration placed over calcium-silicate-based cements.
- The delayed composite restorations, placed after seven days, provided higher shear bond strength than immediate restorations.
- The scanning electron microscopy analysis identified an interdiffusion zone (hybrid layer) between the adhesives and calcium-silicate-based cements, but with differences between the groups. The penetration depth of the adhesives into the cements was higher in the group of immediate adhesive restorations, compared to those performed on the seventh day, and both adhesives penetrated deeper into the NuSmile^®^ NeoMTA, compared to Biodentine^TM^.

## Figures and Tables

**Table 1:** Experimental group details: CSC material, adhesive system, additional hydrophobic bonding layer application and time of adhesive/restorative procedures.

Group	Abbreviation	CSC	Adhesive System	Additional HBL (Clearfil^TM^ SE Bond 2—Bond)	Restoration Time	Restorative Material
1	BiodentineSE0I	Biodentine^TM^	Clearfil^TM^ SE Bond 2	no	12 min	SDR^TM^ Bulk-fill flowable composite
2	BiodentineSE07		Clearfil^TM^ SE Bond 2	no	7 days	
3	BiodentineSE1I		Clearfil^TM^ SE Bond 2	yes	12 min	
4	BiodentineSE17		Clearfil^TM^ SE Bond 2	yes	7 days	
5	BiodentineU0I		Clearfil^TM^ Universal Bond Quick	no	12 min	SDR^TM^ Bulk-fill flowable composite
6	BiodentineU07		Clearfil^TM^ Universal Bond Quick	no	7 days	
7	BiodentineU1I		Clearfil^TM^ Universal Bond Quick	yes	12 min	
8	BiodentineU17		Clearfil^TM^ Universal Bond Quick	yes	7 days	
9	NeoMTASE0I	NuSmile^®^ NeoMTA	Clearfil^TM^ SE Bond 2	no	3 min	SDR^TM^ Bulk-fill flowable composite
10	NeoMTASE07		Clearfil^TM^ SE Bond 2	no	7 days	
11	NeoMTASE1I		Clearfil^TM^ SE Bond 2	yes	3 min	
12	NeoMTASE17		Clearfil^TM^ SE Bond 2	yes	7 days	
13	NeoMTAU0I		Clearfil^TM^ Universal Bond Quick	no	3 min	SDR^TM^ Bulk-fill flowable composite
14	NeoMTAU07		Clearfil^TM^ Universal Bond Quick	no	7 days	
15	NeoMTAU1I		Clearfil^TM^ Universal Bond Quick	yes	3 min	
16	NeoMTAU17		Clearfil^TM^ Universal Bond Quick	yes	7 days	

**Table 2:** Results of the tested groups regarding mean shear bond strength values and fracture patterns concerning main effects comparisons.

Type of Main Effect	Groups	n	SBS Results		*p*-Value	Fracture Pattern Analysis			
			Mean * (SD)	Min/Max		Adhesive	Cohesive CSC	Cohesive RC	Mixed
CSC	Biodentine	160	7.10 (3.91)	0.84/18.48	0.897	45	59	0	56
	NeoMTA	160	7.16 (4.50)	0.85/21.50		38	77	0	45
Adhesive system	CSEB2	160	6.09 (3.86)	0.84/18.22	<0.001	57	66	0	37
	CUBQ	160	8.16 (4.30)	1.78/21.50		26	70	0	64
Additional HBL	No	160	6.58 (4.32)	0.84/21.33	0.014	30	77	0	53
	Yes	160	7.52 (4.03)	0.25/21.50		53	59	0	48
Restoration time	Immediate	160	6.05 (3.49)	0.84/16.66	<0.001	18	96	0	46
	Delayed	160	8.20 (4.59)	0.85/21.50		65	40	0	55

* Mean shear bond strength value (standard deviation—MPa). *p*-value < 0.05 is statistically significant.

**Table 3:** Global results of the tested groups regarding SBS values (MPa).

Groups	n	Mean (SD)	Median (IQR)	Min/Max
1 BiodentineSE0I	20	3.62 (2.78)	2.59 (2.75)	0.84/12.64
2 BiodentineSE07	20	5.85 (2.83)	4.71 (4.64)	1.73/11.19
3 BiodentineSE1I	20	9.19 (4.52)	9.57 (6.99)	2.44/16.34
4 BiodentineSE17	20	7.90 (4.63)	7.55 (7.98)	2.24/16.71
5 BiodentineU0I	20	6.01 (3.31)	4.75 (3.94)	2.01/15.76
6 BiodentineU07	20	9.44 (4.58)	8.55 (7.87)	3.49/18.48
7 BiodentineU1I	20	6.93 (1.94)	6.76 (3.15)	3.18/10.12
8 BiodentineU17	20	7.87 (2.68)	7.50 (3.05)	5.10/16.36
9 NeoMTASE0I	20	4.77 (2.01)	4.76 (3.45)	1.04/7.75
10 NeoMTASE07	20	5.10 (2.17)	5.43 (3.85)	1.98/9.53
11 NeoMTASE1I	20	4.69 (2.29)	4.67 (3.23)	1.23/9.42
12 NeoMTASE17	20	7.65 (5.06)	7.56 (6.94)	0.85/18.22
13 NeoMTAU0I	20	6.49 (4.27)	5.81 (4.39)	2.27/16.66
14 NeoMTAU07	20	11.36 (5.72)	12.04 (9.86)	2.24/21.33
15 NeoMTAU1I	20	6.75 (3.11)	6.08 (4.04)	1.78/13.43
16 NeoMTAU17	20	10.44 (4.65)	10.46 (5.13)	5.28/21.50

**Table 4:** Direct comparison between all the 16 groups (*p*-value).

G	1	2	3	4	5	6	7	8	9	10	11	12	13	14	15	16
1		1.000	<0.001	0.039	1.000	<0.001	0.635	0.044	1.000	1.000	1.000	0.086	1.000	<0.001	1.000	<0.001
2			0.588	1.000	1.000	0.302	1.000	1.000	1.000	1.000	1.000	1.000	1.000	0.001	1.000	0.014
3				1.000	0.890	1.000	1.000	1.000	0.025	0.073	0.020	1.000	1.000	1.000	1.000	1.000
4					1.0300	1.000	1.000	1.000	0.983	1.000	0.812	1.000	1.000	0.434	1.000	1.000
5						0.469	1.000	1.000	1.000	1.000	1.000	1.000	1.000	0.001	1.000	0.024
6							1.000	1.000	0.011	0.033	0.009	1.000	1.000	1.000	1.000	1.000
7								1.000	1.000	1.000	1.000	1.000	1.000	0.025	1.000	0.371
8									1.000	1.000	0.891	1.000	1.000	0.393	1.000	1.000
9										1.000	1.000	1.000	1.000	<0.001	1.000	<0.001
10											1.000	1.000	1.000	<0.001	1.000	0.001
11												1.000	1.000	<0.001	1.000	<0.001
12													1.000	0.215	1.000	1.000
13														0.006	1.000	0.108
14															0.013	1.000
15																0.223

## Data Availability

Data is contained within the article or supplementary material.

## Supplementary Materials

The following are available online at https://www.mdpi.com/article/10.3390/ma14175055/s1, Table S1: Manufacturer, composition, steps for application, lot number of the materials used in the study.
