# Supplementary material for: Evaluation of the Interfaces between Restorative and Regenerative Biomaterials Used in Vital Pulp Therapy
Source: Materials (Basel). 2021 Sep 3;14(17):5055. doi: 10.3390/ma14175055 (PMC8434275; doi:10.3390/ma14175055)
Supplement: Supplementary file 1 [file materials-14-05055-s001.zip › materials-1318620-supplementary.pdf]

materials

MDPI

Supplementary Materials: Evaluation of the Interfaces between Restorative and Regenerative Biomaterials Used in Vital Pulp Therapy

Maria Teresa Xavier [1,*], Ana Luísa Costa [1,2], Francisco José Caramelo [3], Paulo Jorge Palma [2,4] and João Carlos Ramos [5,6]

Table S1. Manufacturer, composition, steps for application, lot number of the materials used in the study.

Material (Batch)	Manufacturer	Classification	Composition	Mode/ Steps of Application
Biodentine™ (B25180)	Septodont, Saint-Maur-des-Fosses Cedex, France	CSC	Powder<br>tricalcium silicate, dicalcium silicate, calcium carbonate and oxide, iron oxide, and zirconium oxide<br>Liquid<br>calcium chloride and hydrosoluble polymer	1. Pour 5 drops of liquid into the capsule.<br>2. Place the capsule on a mixing device.<br>3. Mix for 30 s.
NuSmile® NeoMTA (2019100803)	NuSmile Ltd. Houston, TX, USA	CSC	Powder<br>tricalcium silicate, dicalcium silicate, tantalite, calcium sulfate, tricalcium aluminte<br>Gel<br>Water-based liquid	1. Dispense 1 scoop of powder.<br>2. Dispense one drop of gel.<br>3. Incorporate the gel by spatulating the powder/gel mixture firmly until a putty-like consistency is obtained
Clearfil™ SE Bond 2 Primer (9J01010) Bond (9C0191)	Kuraray Noritake Dental Inc.; Sakazu, Kurashiki, Okayama, Japan	Two-step self-etch adhesive system	Primer<br>MDP, HEMA, hydrophilic aliphatic dimethacrylate, dl-CQ, water<br>Bond<br>MDP, Bis-GMA, HEMA, hydrophobic aliphatic dimethacrylate, dl-Camphorquinone, initiators, accelerators, silanated colloidal silica	1. Apply primer, leave it for 20 s and dry with mild airflow.<br>2. Apply bond and distribute evenly with mild airflow.<br>3. Light cure for 10 s.
Clearfil™ Universal Bond Quick (000018)	Kuraray Noritake Dental Inc.; Sakazu, Kurashiki, Okayama, Japan	One-step self-etch adhesive system	Bond<br>MDP, Bis-GMA, HEMA, hydrophilic amide monomers, colloidal silica, silane coupling agent, sodium fluoride, dl-Camphorquinone, ethanol, water	1. Apply bond and dry the entire cavity wall by blowing mild until the bond does not move.<br>2. Light cure for 10 s.
SDR™ Bulk-fill flowable composite (0217)	Dentsply DeTrey GmbH, Konstanz, Germany	Bulk fill flowable composite	Barium-alumino-fluoroborosilicate glass, strontium alumino-fluoro-silicate glass, modified urethane dimethacrylate resin, EBPADMA, TEGDMA, CQ, photoaccelerator, BHT, UV stabilizer, titanium dioxide, iron oxide pigments, fluorescing agent	1. Dispense SDR™ material.<br>2. Light-curing for at least 20 s.
Ionostar® Molar (19330)	VOCO GmbH, Cuxhaven, Germany	Glass ionomer bulk filling material	Fluoro-aluminosilicate glass, polyacrylic acid, tartaric acid	1. Mix the activated application capsule for 10 - 15 s.<br>2. Apply the material directly into the cavity and it can be worked for at least 1.5 min.<br>3. Finish the restoration with a diamond bur or a polisher and apply a protective varnish
